# Does BRRD mitigate the bank-to-sovereign risk channel?

**DOI:** 10.1371/journal.pone.0292040

**Published:** 2024-04-16

**Authors:** Martien Lamers, Thomas Present, Nicolas Soenen, Rudi Vander Vennet

**Affiliations:** Department of Economics, Ghent University, Ghent, Belgium; Institute for Economic Forecasting, Romanian Academy, ROMANIA

## Abstract

We investigate the effectiveness of the Bank Recovery and Resolution Directive (BRRD) in mitigating the transmission of credit risk from banks to their sovereign, using CDS spreads to capture bank and sovereign credit risk for a sample of 43 banks in 8 Euro Area countries over the period 2009–2020. If the BRRD bail-in framework is credible, changes in bank default risk should not be transmitted to sovereign risk. In a novel approach we use banks earnings announcements to identify exogenous shocks to bank credit risk and investigate to what extent bank risk is transmitted to sovereign risk before and during the BRRD era. We find that bank-to-sovereign risk transmission has diminished after the introduction of the BRRD, suggesting that financial markets judge the BRRD framework as credible. The decline in bank-sovereign risk transmission is particularly significant in the periphery Euro Area countries, especially Italy and Spain, where the bank-sovereign nexus was most pronounced during the sovereign debt crisis. We report that the lower bank-to-sovereign credit risk transmission is associated with the parliamentary approval of the BRRD and not with the OMT program launched by the ECB to affect sovereign yield spreads, nor with specific bail-in or bailout cases which occurred during the BRRD era. Finally, we document that the reduction in risk transmission is most pronounced for banks classified as a Global Systemically Important Bank (G-SIB), stressing the importance of additional capital buffers imposed by Basel III.

## 1. Introduction

Is the BRRD bail-in regime credible? This is an important policy question since the Bank Recovery and Resolution Directive (BRRD) is a key component of the European resolution framework which is designed to organize the orderly unwinding of failing banks without involvement of governments. Reacting to the existential threat of the Euro Area sovereign debt crisis in 2012, the European Council created the Banking Union in an explicit attempt to break the bank-sovereign feedback loop. The European Banking Union consists of three pillars: a Single Supervisory Mechanism (SSM), a Single Resolution Mechanism (SRM) and a European Deposit Insurance System (EDIS). The first pillar is fully operational, the centralized supervision of systemic banks is exercised by the European Central Bank (ECB). The third pillar, a European deposit guarantee scheme is not yet realized, further steps towards a mutualization of national deposit protection schemes is the probable way forward. Here, we focus on the second pillar, the SRM. The framework for the resolution of failing banks and the rules for the bail-in of securityholders in the case of bank distress are governed by the Bank Recovery and Resolution Directive (BRRD). Since 2016, the Single Resolution Board (SRB) is responsible for the application of the BRRD rules, backed up by a Single Resolution Fund which is financed by the banks under the jurisprudence of the SRB. The policy question is: Is the BRRD and the associated bail-in regime considered credible by financial markets? Has it diminished the feedback loop between banks and sovereigns, and more specifically, the transmission of bank risk to sovereign risk?

From a legal and governance perspective, the BRRD and the SRB are fully operational since 2016. Since the resolution of distressed banks is now treated at the European instead of the national level, this should inspire trust by financial markets that the new bail-in regime will be applied effectively. The most widely publicized case was the resolution of Banco Popular in 2017, described by the financial press as a model to deal with failing banks (Financial Times, 8 June 2017). Nevertheless, even in the case of Banco Popular, the subordinated bonds (contingent convertibles) that were supposed to serve as a going concern recapitalization tool, failed to trigger in a timely fashion. The resolution process ended with the SRB transferring ownership of Banco Popular to Banco Santander. Moreover, there have been cases in which sovereigns have intervened to rescue stressed banks, i.e. bailout instead of bail-in. The prime example is Banca Monte dei Paschi di Siena which failed in the 2016 ECB stress test, was subsequently not bailed-in but benefited from a recapitalization by the Italian State in 2017 after invoking the precautionary recapitalization exception clause. As a result, doubts may remain about the willingness of countries to fully apply the bail-in rules [1, 2]. In addition, although resolution may be a workable solution for individual bank distress cases, it remains unclear whether a generalized bail-in would work in a banking crisis affecting many banks simultaneously [3].

The empirical literature investigating the BRRD has generally followed two approaches. One format is event studies investigating the reaction of bank stock returns and CDS spreads around decisions related to the implementation of the BRRD and around actual cases of bank bail-ins. These studies generally report a decrease in investors’ bailout expectations, which suggests that bail-in has become more credible [4–8]. Next to the analysis of specific cases, empirical papers have examined whether or not the BRRD is associated with lower bank-sovereign correlation of bond returns or CDS spreads. Most studies report that the correlation between banks and their sovereign decreased after the introduction of the BRRD [9]. However, [10] conclude that the markets do not judge the BRRD as credible, since instead of the expected widening of the gap between bank and sovereign CDS spreads in the BRRD period, the gap narrows. Some studies also report that although the sovereign/bank correlation decreased, it did not disappear [11]. The correlation between sovereign and bank bond yields or CDS spreads may be caused by the two-way interaction between sovereign risk and bank risk, as shown by [12]. Since the BRRD provides a framework to deal with distressed banks, analyzing sovereign/bank correlations does not allow to judge the credibility of the BRRD since contagion may also flow from sovereigns to banks.

We address the BRRD credibility issue by focusing on the transmission of bank risk to sovereign risk. We argue that the transmission of credit risk from banks to their sovereign is the channel of interest, since the hypothesis that the bail-in framework is credible implies that an increase in bank risk should not be transmitted to higher sovereign risk. To do so, we identify shocks to the bank CDS spreads that are unrelated to the risk of the sovereign by considering the change in bank CDS spreads on quarterly earnings announcement days. Bank earnings of listed banks are disclosed according to a predefined time schedule and their timing is therefore exogenous to bank or sovereign stress. When the announcement is accompanied by an increase in the CDS spread, we consider the announcement as bad news, which increases the risk profile of the bank. Similarly, when the announcement is accompanied by a decrease of the bank-specific CDS spread, the announcement is interpreted as positive news. We then examine whether or not bank default risk is transmitted differently to sovereign risk before and after the implementation of the BRRD.

We acknowledge that the introduction of the BRRD may have lowered the default risk of sovereigns due to the lower probability that they would have to bailout ailing banks and this lower perceived sovereign default risk may spill-over to the banks. However, this effect should materialize when the BRRD is introduced and when the associated bail-in framework is viewed by the CDS market at that moment as credible. Our setup is different. We identify exogenous shifts in the perceived probability of bank distress in the period preceding the BRRD versus the period in which the BRRD is fully operational and analyze whether or not the transmission to sovereign risk has changed. Therefore, the bank-to-sovereign transmission is our channel of interest.

To the best of our knowledge, we are the first to use bank earnings announcements to obtain an exogenous bank credit risk shock. We argue that this setup provides a genuine test of the credibility of the BRRD bail-in framework because we consider bank-specific events in which only bank risk is revealed without a contemporaneous change in sovereign risk. We apply this analysis to 43 banks in 8 Euro Area countries over the period 2009–2020 and find that the bank-to-sovereign risk transmission has diminished in all countries after the introduction of the BRRD. This evidence is consistent with the hypothesis of increased credibility of the BRRD bail-in framework. The decline in bank-sovereign risk transmission is particularly significant in Italy and Spain, where the bank-sovereign nexus was most pronounced during the sovereign debt crisis. We report that the lower bank-to-sovereign credit risk transmission is associated with the parliamentary approval of the BRRD and not with the OMT program launched by the ECB to affect sovereign yield spreads, nor with specific bail-in or bailout cases which occurred during the BRRD era. Finally, we document that the reduction in risk transmission is most pronounced for banks classified as G-SIB, which is consistent with the objective of the BRRD bail-in framework to tackle the too-big-to-fail status of systemic banks. This finding thus provides justification for the additional capital buffers for systemic banks imposed by Basel III.

The paper unfolds in the following way. In Section 2 we review the literature on the BRRD and bail-in. In Section 3 and Section 4 we provide details on the empirical design and the data. Section 5 presents and discusses the main results. Section 6 concludes the paper and formulates policy considerations.

## 2. Related literature

In the literature we find extensive research on the existence of the bank-sovereign feedback loop and the two-way risk spillovers between banks and their sovereign [12–16]. To isolate the bank-to-sovereign transmission or bailout channel, [17] use an instrumental variables approach and report an economically meaningful and highly significant impact of bank sector distress on sovereign distress, whilst [18] find evidence for bank-to-sovereign credit rating spillovers. The transmission from the sovereign to banks, or sovereign-bond channel, is related to the fact that banks typically hold substantial amounts of domestic government bonds on their balance sheet. Several reasons exist for holding bonds of the domestic sovereign. They carry a zero-risk weight in the calculation of risk-weighted assets and thus are not subject to additional capital charges [19], they can be used as collateral against central bank liquidity and for interbank operations [20] or they can be forced upon banks by the domestic sovereign through ‘moral suasion’ [21, 22]. In the European sovereign debt crisis, the interconnections between banks and sovereigns caused severe stress in the banking system and a new round of bailouts. Some even called it the diabolic bank-sovereign loop [23].

Since the introduction of the BRRD, research on the effect of the European resolution framework on the bank-sovereign nexus is receiving increasing attention. In general, theory is ambiguous on the effect of a bank resolution framework on the stability of banks. On the positive side, reducing the likelihood of bailouts and thus taxpayer support, allowing early intervention and providing tools for the orderly resolution of failing banks reduces moral hazard risk [24, 25]. More specifically, the potential of a bail-in and ex-ante knowledge on how losses will be distributed in case of bank failure may increase market discipline. They can also reduce incentives for banks to build up leverage in their balance sheets [26, 27]. On the other hand, a rule-based resolution system can result in bank runs [28] and contagion which would render the banking system less stable because of the direct interlinkages between banks and the possibility of a sudden reassessment of bank risk [29, 30]. According to this view, bailouts of failing banks can protect other banks from contagion and thus provide incentives to reduce risk-taking [31, 32].

Turning to the case of the European resolution framework, [9] empirically examine the effect of changes in sovereign CDS spreads on bank CDS spreads in three periods surrounding the introduction of the BRRD. They report that the sensitivity of bank CDS spreads for changes in sovereign CDS spreads diminishes in subperiods after the introduction of the BRRD, suggesting that the new bail-in regime decreased the interconnections between sovereigns and banks. Similarly, studies investigating the correlation between sovereign and bank CDS spreads typically conclude that it has diminished in the BRRD era, although it has not become insignificant [11]. However, the mitigation of the bank-sovereign nexus may be caused by lower bank risk, but also by lower sovereign risk. [33] argue that accommodative ECB monetary policy is associated with lower bank CDS spreads and that a large part of this effect is caused by lower sovereign CDS spreads. Hence, the diminished interconnection between sovereign and banks may be caused by lower sovereign risk causing decreased bank credit risk. We try to isolate the bank-to-sovereign risk transmission channel in our empirical design, because the BRRD is designed to avoid bank distress spilling over to the sovereign.

Another strand of the empirical BRRD literature uses event studies around important dates for the BRRD implementation process or around actual bail-in cases. Studies by [4–8] focus on investors’ reactions to regulatory and bail-in announcements and usually report a decrease in investors’ bailout expectations, which suggests that bail-in has become more credible. [4] estimate the stock price and CDS reaction to actual bail-ins (e.g. the Cypriot banks and the Portuguese Banco Espirito Santo) and to announcements related to the introduction of the BRRD. The authors find an increase in CDS spreads suggesting that the new regime reduces bailout expectations. Comparable results for stock prices have been reported by [7] examining a broader set of regulatory announcements. [5] considers the difference in yields between banks’ bail-inable bonds and non-bail-in bonds. The main finding is that the spread between both types of bonds increases after events signaling an increased commitment by authorities to bail-in (again based on effective bail-in cases and regulatory announcements). In a similar approach, [6] study the difference in yield between bail-inable and non-bail-in bonds for Italian banks, before and after the introduction of the bail-in tool in January 2016 and conclude an increase of the spread at issuance. Using the same cut-off date, [8] finds a bail-in premium for unsecured bonds maturing after January 2016 compared to similar bonds maturing before that date.

**Fig 1:**
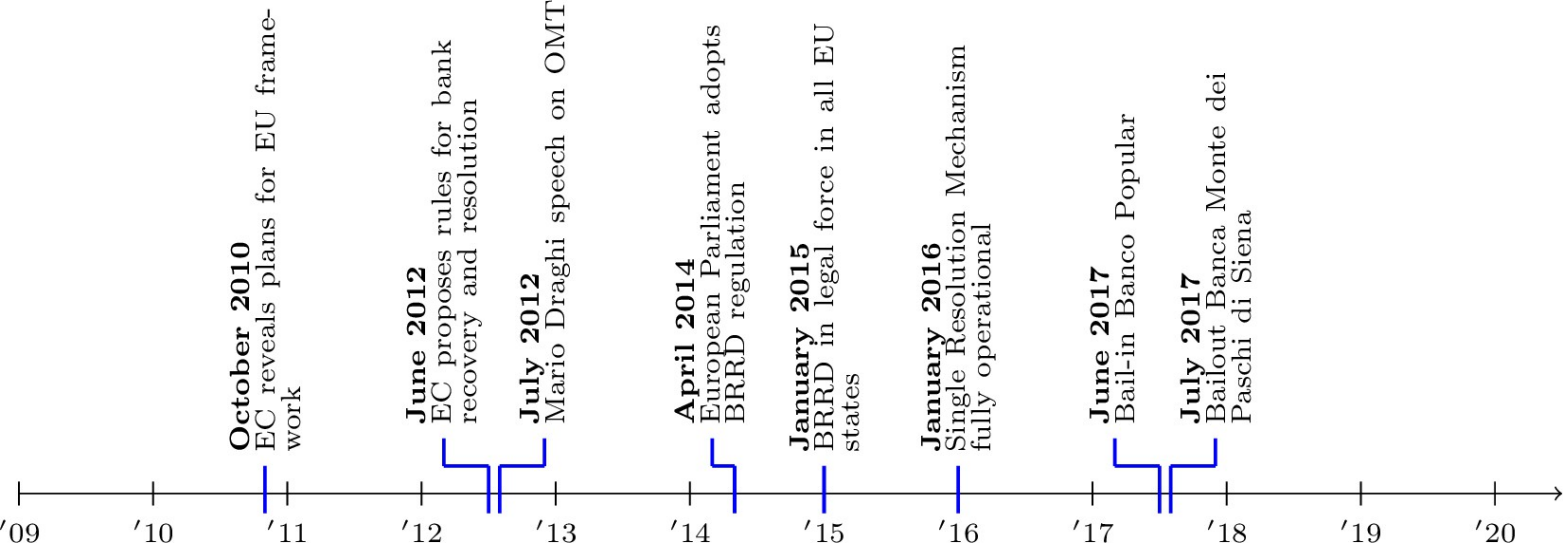
This figure gives a chronological overview of the different events during the sample period of 2009–2020 that are related to the implementation of BRRD or that might have an impact on the transmission of risk between sovereigns and the banking sector.

Yet, some studies cast doubt over the credibility of the BRRD bail-in framework. [10] use a difference-in-differences approach with banks as the treated group and non-financial corporations as the control group. Instead of the expected widening of the gap between bank and sovereign CDS spreads in the BRRD period, the gap narrows, implying that bail-in is not credible. Interestingly, they report that the strongest credibility of the new regime is revealed in Italy. [34] exploit a 2014 change in the definition of credit default swaps for European banks to show that the market price of protection against losses from government interventions exhibited a downward trend from 2014 to 2016, but this trend reversed, indicating a reversal of credibility of the bail-in instrument. A feature of some of the empirical papers is that they rely on the timing of the BRRD implementation to conduct their analyses. Yet, determining when the markets consider the BRRD to be effective is not straightforward. In fact, as is shown in Fig 1, the European Commission revealed its plans for a new EU framework for crisis management in the banking sector in October 2010, it proposed rules for bank recovery and resolution in June 2012, the European Parliament adopted the BRRD on 15 April 2014 and finally, the BRRD entered into legal force in all EU member states on 1 January 2015, while the Single Resolution Mechanism became fully operational on 1 January 2016. Hence, determining exact dates for event studies is difficult, also because such regulation is often anticipated by financial markets [35].

We contribute to the literature by focusing on the transmission of credit risk from banks to their sovereign. Since the BRRD is designed to avoid contagion of increased credit risk from distressed banks to their sovereign via bailouts, the bank-to-sovereign risk transmission is the channel of interest to establish whether or not investors judge the BRRD as credible. To do so, we identify shocks to the bank CDS spreads that are unrelated to the risk of the sovereign by considering the change in bank CDS spreads on quarterly earnings announcement days. Other approaches have been implemented to detect spillover from banks to sovereigns, e.g. using non-Eurozone bank stock prices as instruments [17] or using bank and sovereign credit ratings [18]. We isolate the bank-to-sovereign credit risk channel by identifying bank risk shocks on earnings announcement days, which are exogenous because they follow a predetermined time schedule. Another contribution of the paper is that we test the robustness of the choice of the timing of the BRRD introduction. As Fig 1 indicates, the implementation of the BRRD has been a staggered process. The crucial date is 2016 since the BRRD framework is in full legal force since that year. However, the BRRD received parliamentary approval in April 2014, hence market participants could anticipate the full implementation of the BRRD legal framework. Instead of imposing an introduction timing([36] argue that 2014 is the relevant date while [10] use 2016), we experiment with both and also conduct a year-by-year analysis to investigate the time-varying pattern of the bank-to-sovereign risk transmission channel. Fig 1 also identifies events that may have an effect on sovereign risk (the Draghi ‘whatever it takes’ speech in 2012) or affect the market perception of the credibility of bail-in (the treatment of Banco Popular and Banca Monte dei Paschi di Siena in 2017), we include these in our analysis. Finally, we contribute by analyzing the bank-to-sovereign nexus for core versus periphery Euro Area countries and by linking the bank-sovereign transmission intensity to the systemic nature (G-SIB status) of the banks.

## 3. Data and sample selection

Since we want to analyze the impact of changes in bank credit risk on sovereign risk, we use Credit Default Swap (CDS) spreads since they capture the market consensus about default risk. Compared to bank and sovereign bond spreads, CDS spreads have three main advantages. First, CDS spreads provide timelier market-based pricing [37, 38]. Second, using CDS spreads avoids the difficulty in dealing with time to maturity as in the case of using interest rate spreads (of which the zero coupon bonds would be preferred). Third, bond spreads include inflation expectations and demand/supply conditions as well as default risk. Since we explicitly want to single out default risk or tail risk, we focus on CDS spreads [13]. We argue this is the relevant risk measure to analyze when estimating the bank-to-sovereign risk transmission channel, since bailouts/bail-ins occur when banks are failing or likely to fail.

Consequently, we select all the banks with outstanding CDS contracts. For both banks and sovereigns we use the CDS spreads on 5-year senior bonds, because they are the most liquid type of CDS. We retrieve CDS data from IHS Markit. For the banks we use the Modified-modified restructuring (MM) contractual term clause. For the sovereigns we use the Full or Complete Restructuring (CR) term clause. We include all banks that have a CDS spread quotation in at least 25% of the daily observations over the period 2009–2020, resulting in a sample of daily CDS rates for 43 banks in 8 Euro Area countries. The resulting list of banks is shown in the Table A.1 in S1 Appendix. Due to the Greek sovereign debt restructuring in March 2012, the CDS spreads for Greece are unavailable from March 2012 to June 2013. As this is an important period closely before the approval of BRRD, it was excluded from our analyses. Although the sample is limited to banks with 5-year CDS contracts, these are typically the larger banks, which are the main targets of the BRRD bail-in rules.

**Fig 2:**
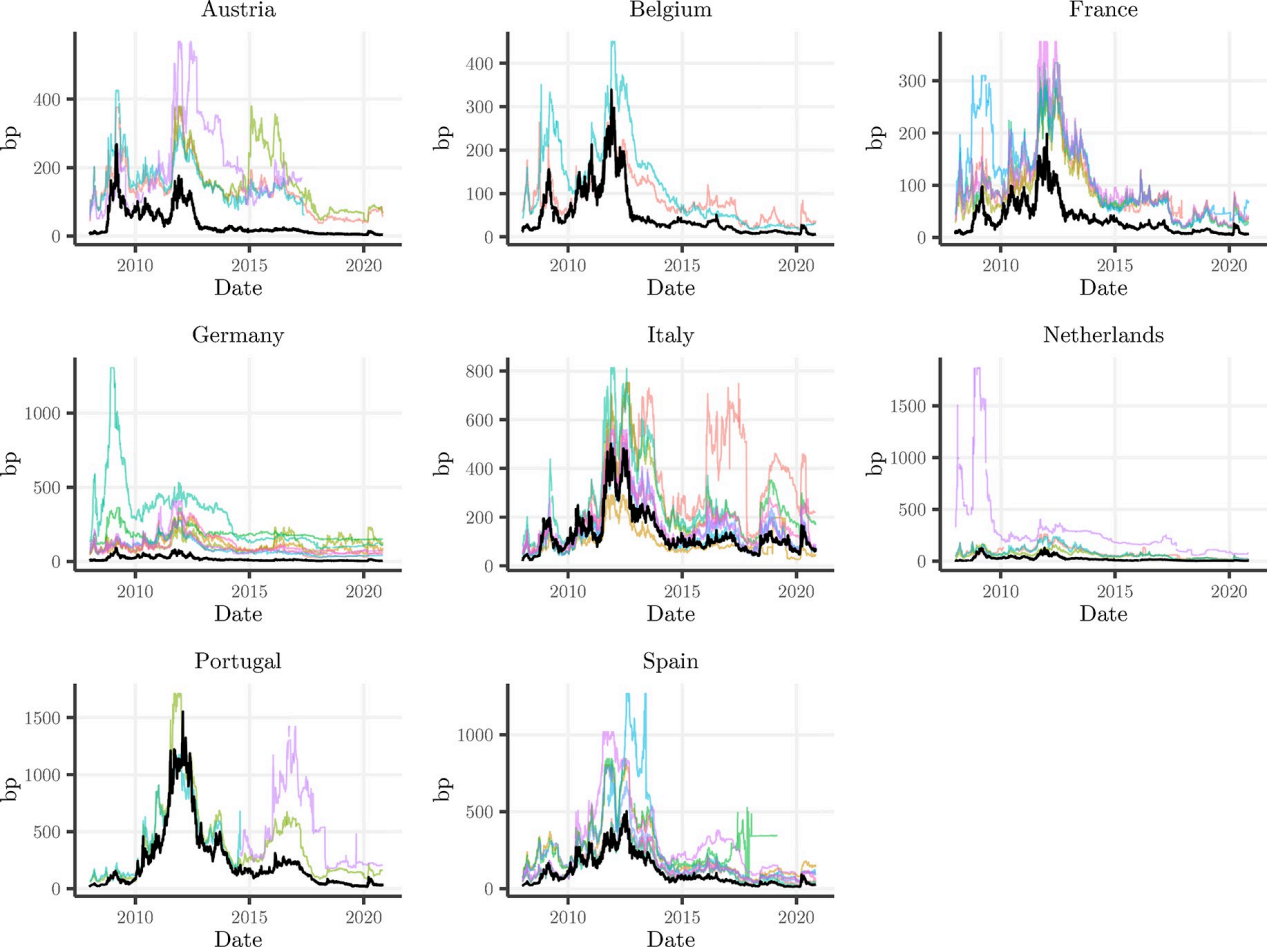
This figure shows the time series of the CDS spread of the banks and sovereigns in the sample grouped by country. Colored lines represent individual banks and black lines represent the local sovereign. The vertical axes are not aligned over different countries to allow the CDS spread of all banks to be fully captured. Legends for the bank CDS series are omitted in order not to overload the figures. Some notable examples are: UniCredit Bank Austria (AT, pink), Raiffeisen Bank International (AT, lime), Natixis (FR, blue), IKB Deutsche Industriebank (DE, teal), Deutsche Bank (DE, khaki), Banca Monte dei Paschi di Siena (IT, red), NIBC bank (NL, pink), Novo Banco (PT, pink), Bankia (ES, teal), Banco Popular Español (ES, green).

Fig 2 displays the evolution of the CDS spread for each bank in our sample, accompanied by the sovereign CDS spread in black. We can discern periods of heightened CDS spreads with respect to both banks and sovereigns. The global financial crisis followed by the sovereign debt crisis led to significant increases in perceived bank and sovereign default risk. But also in the post-crisis period, some banks in some countries exhibit spikes in their CDS spreads, indicating stress and rising market doubts about their creditworthiness. Finally, in the first half of 2020 we notice elevated levels due to the onset of the Covid pandemic.

In our estimations we control for bank and country characteristics and macroeconomic dynamics. We capture the risk profile of a bank with its unweighted capital ratio. The systemic importance of a bank is captured by the ratio of its total assets to the GDP of the home country and their G-SIB buffer. These data are obtained from S&P Capital IQ Pro, Eurostat and Refinitiv. The macroeconomic environment in Europe is controlled for by including the Vstoxx and Stoxx Europe 600 and sovereign indebtedness is captured by the Debt/GDP ratio, retrieved from Eurostat and Refinitiv. Finally, to control for broad credit risk in the European banking sector, we employ the iTraxx Financials CDS index, obtained from Refinitiv, which represents the average level of the CDS of the 30 largest financial institutions in Europe.

The descriptive statistics of the bank and sovereign CDS spreads, bank and country control variables and market variables are reported in Table 1, and the correlation matrix is reported in Table 2.

**Table 1:** Descriptive statistics for the bank and sovereign CDS spreads, the bank and country variables and the market variables.

Variable	Mean	Std	P1	Median	P99
CDS Bank	173.26	172.49	21.25	121.75	891.56
CDS Sovereign	84.51	125.58	4.47	42.50	501.52
Capital	6.30	2.70	1.54	6.27	14.09
Bank Total Assets / GDP	35.29	33.92	0.48	22.53	141.47
Sovereign Debt / GDP	92.49	27.12	36.40	90.60	149.20
Itraxx Financials	178.70	108.13	56.12	140.39	499.17
G-SIB Buffer	0.20	0.48	0.00	0.00	2.00
Stoxx Europe 600	141.05	41.81	64.89	145.10	216.82
Vstoxx	23.68	9.72	11.66	21.80	60.71

**Table 2:** Correlation matrix.

Name	CDS Bank	CDS Sovereign	Capital	Bank Total Assets/GDP	Sovereign Debt/GDP	Itraxx Financials	G-SIB Buffer	Stoxx Europe 600	Vstoxx
CDS Bank	1								
CDS Sovereign	0.72	1							
Capital	-0.01	0.03	1						
Bank Total Assets/GDP	-0.17	-0.06	-0.18	1					
Sovereign Debt/GDP	0.16	0.31	0.20	-0.14	1				
Itraxx Financials	0.54	0.54	-0.21	0.05	-0.06	1			
G-SIB Buffer	-0.14	-0.09	-0.26	0.59	-0.10	0.04	1		
Stoxx Europe 600	-0.31	-0.33	0.25	-0.08	0.26	-0.68	-0.04	1	
Vstoxx	0.18	0.19	-0.09	0.05	-0.16	0.38	0.01	-0.56	1

## 4. Empirical specification

We test whether or not the bank-to-sovereign risk transmission is affected by the implementation of the BRRD. To do so we estimate the following regression on bank earnings announcement days, i.e. days on which potentially new information about the risk profile of the banks is disclosed:

$${\Delta CDS}_{i,t}^{sov}=\beta_{1}{\Delta BankRisk}_{i,j,t}+\beta_{2}{BRRD}_{t}+\beta_{3}{\Delta BankRisk}_{i,j,t}\times {BRRD}_{t}+\sum_{k=1}^{K}\vartheta_{k}{Controls}_{k,j,t}+\varepsilon_{j,t}$$ (1)

where ${\Delta CDS}_{i,t}^{sov}$ is the first difference of the sovereign CDS spread of country *i* on earnings announcement day *t*. Δ*BankRisk*_*i*,*j*,*t*_ is the first difference of the credit risk of bank *j* located in country *i* on earnings announcement day *t*. We control for the bank, country and macroeconomic variables outlined in Section 3. Since the regression is estimated in first differences we choose not to include country fixed effects as CDS spreads are generally assumed to be stationary over a longer period of time. In order to obtain the heterogeneous impact of bank credit risk on sovereign credit risk before and after the implementation of BRRD, we interact the bank credit risk variable with a BRRD implementation dummy variable (*BRRD*_*t*_). Given the staggered implementation of the BRRD (see Fig 1) we argue that two dates are of particular importance. The first date is April 2014 since this is the time when European Parliament approved the BRRD and this should have provided certainty to market participants that the BRRD is the new legal framework to deal with distressed banks. The second is 2016 since the BRRD entered into full legal force in January 2016. From that moment onwards, the BRRD rules are legally binding in all countries. Consequently we test the effectiveness of the BRRD with a BRRD dummy which is 0 for the period before April 2014 and 1 afterwards, alternatively with a BRRD dummy equal to 1 from January 2016 onwards and also with both dummies included. If BRRD is effective it should reduce the transmission of bank credit risk to sovereign risk, implying that the coefficient *β*_3_ should be significantly negative.

As highlighted in Section 1 the econometric identification of Eq (1) is vulnerable to omitted variable and reverse causality biases [17]. The omitted variable bias originates from the fact that both sovereign and bank credit risk may be driven by general economic dynamics. Therefore we include the evolution of the Stoxx Europe 600 stock market index and the Vstoxx as a measure of financial market volatility in all estimations. Moreover, in order to conduct a genuine investigation of the bank-to-sovereign risk channel, we need to isolate bank risk shocks unrelated to changes in the general risk perception of the Euro Area banking sector. To address this we obtain a measure of risk that can be attributed to each bank by running the following regression:

$${\Delta CDS}_{j,t}^{bank}=\beta_{0}+\beta_{1}{\Delta iTraxxFinancials}_{t}+\varepsilon_{j,t}$$ (2)

**Fig 3:**
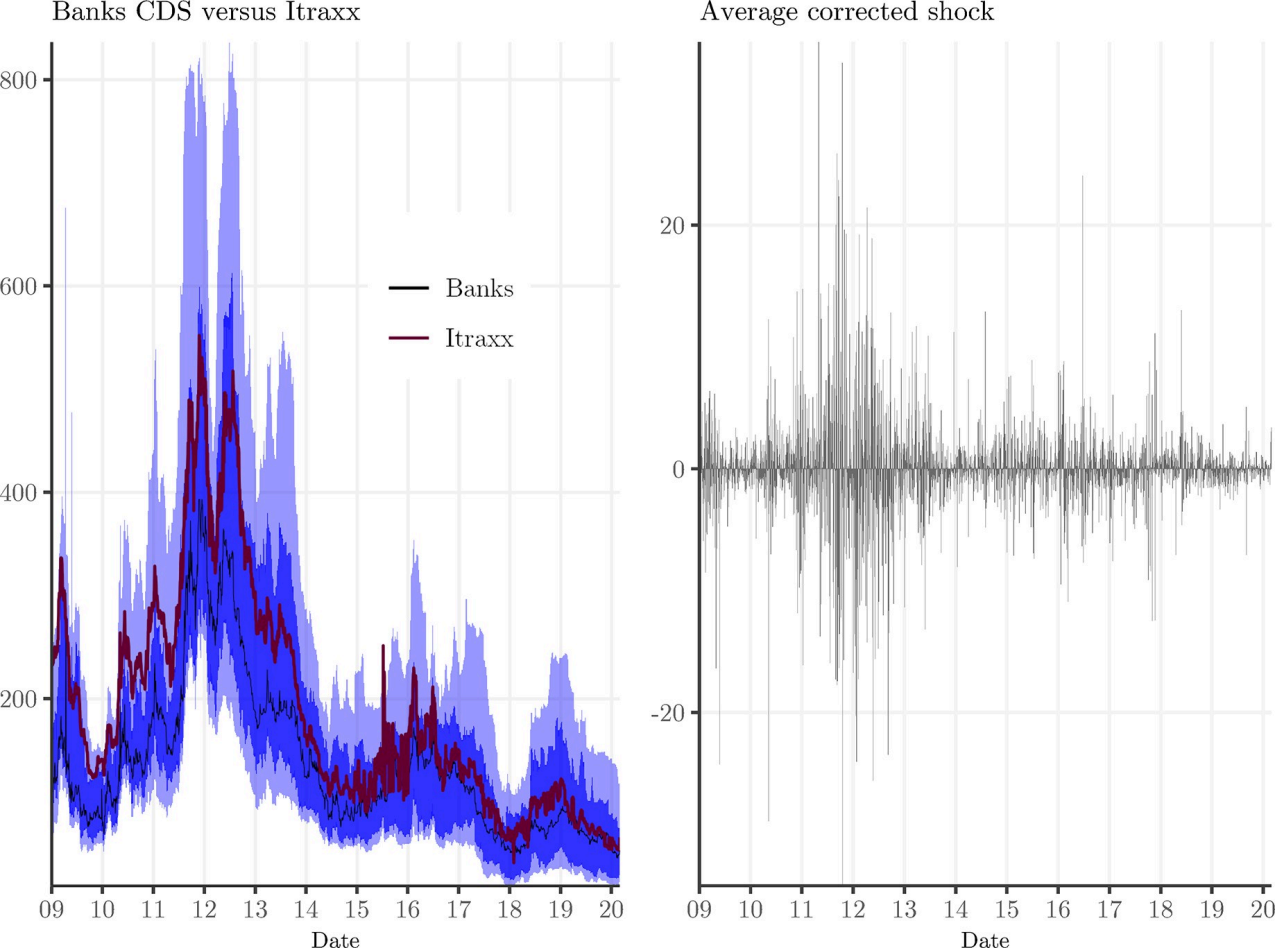
Descriptives of the exogenous bank risk shock on earnings announcement days. In the left panel we show the time series of both bank CDS spreads and the iTraxx Financials CDS index. The solid black line represents the median value of bank CDS spreads, while the darker and lighter blue areas represent the 25–75% and the 10–90% percentiles. The iTraxx is shown in red. In the right panel we show the evolution of the average bank shock over time, which suggests that bank credit risk shocks were more volatile during the sovereign debt crisis.

Where Δ*iTraxxFinancials*_*t*_ is the change of the iTraxx Financials CDS index on day *t*. This setup is similar to a CAPM estimation for stock returns, where a broad market index is used to derive the *β* of a portfolio or individual stock. The variation that is not explained by the market index is the ‘abnormal’ stock return. We run this regression for each bank prior to each earnings announcement and obtain the ‘abnormal’ CDS spread change as the out-of-sample prediction of $\hat{\varepsilon_{j,t}}$, which we denote Δ*BankRisk*_*i*,*j*,*t*_. We use an estimation window of 250 days and a buffer period of 10 days before the earnings announcement. This ‘abnormal’ CDS spread change in Eq (1) is orthogonal to the general market evolution captured by the iTraxx Financials CDS index. In Fig 3 we give an overview of the estimated parameters and the average value of the bank default risk shock.

To deal with the problem of reverse causality, we estimate Eq (1) only on bank earnings announcement days. These are the days on which bank-specific information is released which may affect the perception of the bank’s risk profile. The number of earnings announcement days per bank in our sample is shown in the last column of Table A.1 in S1 Appendix. The advantage of using earnings announcement days is that an earnings disclosure calendar is determined long in advance, which ensures that the timing of the announcement cannot endogenously be influenced by changes in sovereign credit risk. The ‘abnormal’ change of the CDS spread on the earnings announcement day thus captures the unanticipated component in the earnings release and therefore represents an exogenous shock to underlying bank credit risk. An increase of the bank-specific CDS spread on earnings announcement days is interpreted as a negative news event and vice versa. [39] show that changes of CDS spreads are able to capture the impact of earnings surprises, as well as anticipation effects on the earnings surprise prior to its announcements. Hence, the change in banks’ CDS spreads on earning announcements days are reliable indicators of exogenous bank shocks. We do not classify bank earnings announcements as positive or negative news depending on the deviation of the announced earnings per share from the expectations of analysts, because the quarterly disclosures do not only contain information about the banks’ profits, but in most cases also reveal information about, e.g. dividends, management changes, management expectations about costs and revenues or the impact of macroeconomic developments, which may materially impact the banks’ risk profile. We assume that the combined effect of these announcements as perceived by investors are captured by the changes in the banks’ CDS spreads.

## 5. Results

The results of estimating Eq (1) with BRRD dummies starting in 2014 and/or 2016 are reported in panels A, B and C of Table 3. In columns (2) and (3) we add bank, country and macro controls for heterogeneity in bank risk, sovereign indebtedness and macroeconomic dynamics. The bank, country and macro controls are not reported in the tables in order not to overload them. The results are mostly insignificant. Where the results do turn out to be significant, their interpretation is in line with our assumptions. In column (4) we add country and year fixed effects to control for unobserved country and time variation. The first row shows the impact of a shock to bank credit risk prior to the introduction of the BRRD, whilst the second row captures the change in the transmission of bank to sovereign default risk since the implementation of the BRRD. The results with the 2014 BRRD dummy indicate that in the period of the sovereign debt crisis and before the introduction of BRRD, around 41–45% of a shock to the bank CDS is transmitted towards its home sovereign. This result is in line with results found in the literature using different methodologies in a similar sample period (e.g. [12] find a transmission of between 18–49%). After the BRRD was approved by the European Parliament, this transmission has diminished significantly by 34–38%points. This result suggests that the BRRD has been effective in diminishing the bank-sovereign risk nexus at the Euro Area level. This result is confirmed when we estimate the shock in a 2-day event window, which controls for delayed reactions of bank risk shocks. Most banks typically disclose their quarterly statements before the opening of financial markets, but some also after-market closure. We include the results in Table B.1. in the S1 Appendix. When we use 2016 as the BRRD implementation moment in Panel B, we find that prior to the full entry into force of the BRRD, around 39–43% of a bank shock is transmitted to its home sovereign. After the BRRD introduction, the bank-to-sovereign risk transmission reduced significantly with 29–33% points. This raises the question which of the two implementation dates is considered by the markets as the decisive one. Panel C shows that the decline of the bank-to-sovereign risk transmission is fully caused by the parliamentary approval of the BRRD in 2014, the full entry into legal force of the BRRD in 2016 does not diminish the transmission channel further. Hence, market participants considered the BRRD credible from the moment it was enacted by the Parliament.

**Table 3:** Estimation of the transmission of bank-to-sovereign credit risk using dummies for the introduction of the BRRD regulation. Panel A displays the results for the BRRD dummy from 15/04/2014 onwards, panel B shows the findings when considering 01/01/2016 as the BRRD implementation. Standard errors in parentheses are clustered at the bank level. *** represents significance at the 1% percent level.

Panel A: BRRD approved on 15/04/2014				
	(1)	(2)	(3)	(4)
	Δ*CDS*^*sov*^	Δ*CDS*^*sov*^	Δ*CDS*^*sov*^	Δ*CDS*^*sov*^
Δ*BankRisk*	0.45∗∗∗	0.45∗∗∗	0.41∗∗∗	0.45∗∗∗
	(0.11)	(0.11)	(0.11)	(0.10)
Δ*BankRisk*×*BRRD*	-0.34∗∗∗	-0.34∗∗∗	-0.38∗∗∗	-0.35∗∗∗
	(0.10)	(0.10)	(0.10)	(0.10)
Bank and Country Controls	No	Yes	Yes	Yes
Macro Controls	No	No	Yes	No
Country and Year FE	No	No	No	Yes
R^2^	0.12	0.12	0.16	0.15
Adj. R^2^	0.12	0.11	0.15	0.14
No. Obs.	1,545	1,543	1,543	1,543
Panel B: BRRD effective from 01/01/2016				
	(1)	(2)	(3)	(4)
	Δ*CDS*^*sov*^	Δ*CDS*^*sov*^	Δ*CDS*^*sov*^	Δ*CDS*^*sov*^
Δ*BankRisk*	0.43∗∗∗	0.42∗∗∗	0.39∗∗∗	0.43∗∗∗
	(0.09)	(0.10)	(0.09)	(0.09)
Δ*BankRisk*×*BRRD*	-0.30∗∗∗	-0.29∗∗∗	-0.33∗∗∗	-0.31∗∗∗
	(0.09)	(0.09)	(0.09)	(0.08)
Bank and Country Controls	No	Yes	Yes	Yes
Macro Controls	No	No	Yes	No
Country and Year FE	No	No	No	Yes
R^2^	0.16	0.11	0.15	0.15
Adj. R^2^	0.14	0.11	0.15	0.13
No. Obs.	1,545	1,543	1,543	1,543
Panel C: BRRD approval and implementation				
	(1)	(2)	(3)	(4)
	Δ*CDS*^*sov*^	Δ*CDS*^*sov*^	Δ*CDS*^*sov*^	Δ*CDS*^*sov*^
Δ*BankRisk*	0.45∗∗∗	0.45∗∗∗	0.41∗∗∗	0.45∗∗∗
	(0.11)	(0.11)	(0.11)	(0.10)
Δ*BankRisk*×*BRRD*_2014_	-0.40∗∗∗	-0.39∗∗∗	-0.41∗∗∗	-0.37∗∗∗
	(0.10)	(0.10)	(0.10)	(0.12)
Δ*BankRisk*×*BRRD*_2016_	0.08∗	0.08∗	0.04	0.04
	(0.05)	(0.05)	(0.04)	(0.05)
Bank and Country Controls	No	Yes	Yes	Yes
Macro Controls	No	No	Yes	No
Country and Year FE	No	No	No	Yes
R^2^	0.12	0.12	0.16	0.15
Adj. R^2^	0.11	0.11	0.15	0.14
No. Obs.	1,545	1,543	1,543	1,543

Establishing that the bank-sovereign risk channel has diminished after the introduction of the BRRD does not imply that it has disappeared. Hence, we examine whether or not the bank-sovereign risk nexus still exists after the introduction of the BRRD, i.e. whether there is still a significant transmission of bank risk to the sovereign. Therefore, we adjust the baseline model in Eq (1) such that we are able to determine whether or not the transmission of the bank-sovereign risk nexus is still significant after the introduction of the BRRD. We estimate the following specification:

$${\Delta CDS}_{i,t}^{sov}=\beta_{1}{\Delta BankRisk}_{i,j,t}\times Pre-{BRRD}_{t}+\beta_{2}{\Delta BankRisk}_{i,j,t}\times {BRRD}_{t}+\beta_{3}{Pre-BRRD}_{t}+\beta_{4}{BRRD}_{t}+\sum_{k=1}^{K}\vartheta_{k}{Controls}_{k,j,t}+\varepsilon_{j,t}$$ (3)

Where *Pre ‒ BRRD*_*t*_ is a dummy variable which equals 1 before the introduction of the BRRD and 0 afterwards. This adjustment leads to a different interpretation of *β*_2_. Whilst in Eq (1) this coefficient captures the change in the transmission of bank risk to the sovereign after the introduction of the BRRD, this now captures the total transmission of bank to sovereign default risk when the BRRD is in place. In Eq (3), *β*_1_ captures the transmission of bank to sovereign default risk prior to the introduction of the BRRD. Table 4 shows the results with the 2014 BRRD dummy. In the first two columns, the transmission of bank to sovereign credit risk appears to be still significant after the implementation of BRRD. However, when gradually saturating the model with bank-specific, country and macroeconomic controls, we no longer find any significant impact of bank credit risk shocks on sovereign CDS spreads, implying that BRRD has been effective in mitigating the transmission channel. In our preferred specification, with bank, country and macro controls, column (3) demonstrates that the bank-to-sovereign credit risk transmission channel was reduced from more than 40% before the BRRD was enacted, to 10% or lower thereafter. Hence, the enactment of the BRRD succeeded in all but eliminating the transmission of exogenous bank default risk shocks to their sovereign.

**Table 4:** Estimation of the bank-to-sovereign risk transmission before and during the BRRD. The table displays the findings when considering 15/04/2014 as the BRRD implementation. Standard errors in parentheses are clustered at the bank level. *** represents significance at the 1% percent level.

	(1)	(2)	(3)	(4)
	Δ*CDS*^*sov*^	Δ*CDS*^*sov*^	Δ*CDS*^*sov*^	Δ*CDS*^*sov*^
Δ*BankRisk*×*Pre*‒*BRRD*_*t*_	0.45∗∗∗	0.45∗∗∗	0.42∗∗∗	0.45∗∗∗
	(0.11)	(0.11)	(0.10)	(0.11)
Δ*BankRisk*×*BRRD*_*t*_	0.11∗∗∗	0.11∗∗∗	0.04	0.10∗∗∗
	(0.03)	(0.03)	(0.04)	(0.03)
Bank and Country Controls	No	Yes	Yes	Yes
Macro Controls	No	No	Yes	No
Country and Year FE	No	No	No	Yes
R^2^	0.12	0.12	0.16	0.15
Adj. R^2^	0.12	0.12	0.16	0.14
No. Obs.	1,545	1,537	1,537	1,537

### 5.1 Time variation, country heterogeneity, and bank characteristics

When analyzing the impact of the BRRD, a number of natural extensions of the impact analysis may provide deeper insight: exploring country heterogeneity and assessing the impact of bank and country characteristics. But first we further examine the issue of time variation in the bank-sovereign risk channel in order to complement the dummy approach.

### 5.2 Timing of the BRRD implementation and the effect of other events

As already indicated in Fig 1, the BRRD has been proposed and implemented over the course of multiple years. Using dummies is the natural approach to distinguish a pre-BRRD and BRRD period and we argued that markets consider 2014 (parliamentary approval) rather than 2016 (full legal force) as the most relevant benchmark date. Yet, choosing a single date to analyze the effect of the BRRD on the transmission channel may be arbitrary. Therefore, we also take an agnostic approach with respect to the timing by simply estimating the impact of BRRD on the bank-sovereign risk nexus on a year-by-year basis. This should reveal if and when financial markets considered the BRRD credible. The specification is:

$${\Delta CDS}_{i,t}^{sov}=\sum_{l=1}^{L}\beta_{l}{\Delta BankRisk}_{i,j,t}\times {YEAR}_{l}+\sum_{k=1}^{K}\vartheta_{k}{Controls}_{k,j,t}+\varepsilon_{j,t}$$ (4)

**Fig 4:**
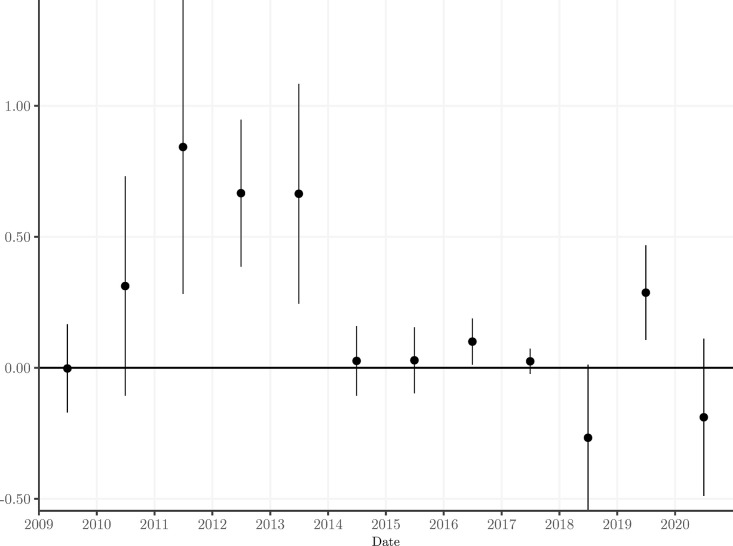
This figure plots the year-by-year evolution of the effect of bank credit risk shocks on sovereign credit risk. The vertical axis denotes the size of the beta coefficients of the interactions of the bank shocks with the year dummies. The 5^th^ to 95^th^ percentile confidence interval is plotted as a vertical bar around the estimated beta.

Where *YEAR*_*l*_ is a dummy variable equal to 1 during year *l* and 0 otherwise. Hence, this model shows the full transmission of bank risk towards the risk of the sovereign in each year separately. The results are shown in Fig 4. Several observations warrant attention. During the sovereign debt crisis, there is a clear increase of the bank-to-sovereign risk channel, confirming similar findings in the literature. In terms of the timing of the decrease of the bank-sovereign risk channel, we observe that the largest decline occurs in 2014, which coincides with the approval of the BRRD by the European Parliament. This finding is consistent with our results reported in Tables 3 and 4. Market participants were convinced that the new bail-in rules were credible from that moment onwards. The bank-sovereign risk channel remains insignificant for the Euro Area in the subsequent period of full implementation of the BRRD, including its entry into full legal force in 2016. Also important to notice is that we do not observe a decline in the bank-sovereign nexus prior to 2014, hence actions such as the ECB’s Outright Monetary Transactions (OMT) program did not visibly weaken the negative spill-overs from bank to sovereign risk. This confirms that OMT succeeded in lowering the spreads of distressed sovereigns, but did not necessarily mitigate bank-to-sovereign risk spillovers. The somewhat erratic behavior of the bank-sovereign channel at the end of the sample period does not appear to be linked to specific events.

Having established that the parliamentary approval of the BRRD has significantly diminished the transmission of bank default risk to sovereign credit risk, a remaining concern is that this effect may be related to monetary policy actions, especially ECB actions designed to tackle the Eurozone debt crisis. Moreover, actual bail-ins of ailing banks or, alternatively, the non-application of the BRRD rules may affect the market’s perception of the enforcement of the BRRD. Therefore, we subdivide our sample period into regimes, demarcated by OMT, BRRD and the bail-in/bailout events in 2017.

The first event of interest is the July 2012 ‘Whatever it takes’ speech by ECB president Mario Draghi in anticipation of the launch of the OMT program aimed at supporting stressed sovereigns. The second event is the treatment of distressed banks in 2017, the bail-in of Banco Popular Espanol (BPoP) in Spain and the rescue of Banca Monte dei Paschi di Siena (BMPS) in Italy. The ‘Whatever it takes’ speech was successful in lowering sovereign spreads in the periphery Euro Area countries [40–42]. However, OMT was targeted at lowering sovereign bond spreads, hence we expect that this monetary policy measure will affect the sovereign-to-bank transmission of credit risk, but we do not expect it to have a significant impact on our channel of interest from banks to sovereigns. The BPoP and BMPS cases are bank-specific events and examples of the diverging treatment of stressed banks. Hence, they may affect the market perception of the general applicability of the BRRD rules. Yet, although the BPoP resolution by the SRB was described as successful, the bail-in framework was not applied in full, since two outstanding CoCo’s of BPoP failed to be triggered in going concern because at the time of resolution the banks still exhibited a CET1/RWA ratio above the CoCo thresholds. As a result, it can be argued that the BRRD rules were not applied in full. This caveat applies in an even stronger fashion to the treatment of BMPS. Instead of putting the bank in resolution after it failed the 2016 EBA/ECB stress test, the Italian government elected to invoke the exception clause of a precautionary recapitalization foreseen in the BRRD. Hence, the two cases were treated differently and this may have consequences for the perceived credibility of the BRRD. Based on these arguments, we do not expect to observe a significant decrease of bank-sovereign risk transmission after these two bank distress cases, especially in Spain and Italy. To test this conjecture, we estimate Eq (1) on earnings announcement days including interactions with a dummy that is one for the following events: *Post‒Draghi*_*t*_ is 1 as of July 26, 2012; *Post‒Bailin*_*t*_ is 1 as of June 7, 2017.


$${\Delta CDS}_{i,t}^{sov}=\beta_{1}{\Delta BankRisk}_{i,j,t}+\beta_{2}{\Delta BankRisk}_{i,j,t}\times {Draghi/OMT}_{t}+\beta_{3}{\Delta BankRisk}_{i,j,t}\times {BRRD}_{t}+\beta_{4}{\Delta BankRisk}_{i,j,t}\times {Bailin}_{t}+\sum_{k=1}^{K}\vartheta_{k}{Controls}_{k,j,t}+\varepsilon_{j,t}$$ (5)

**Fig 5:**
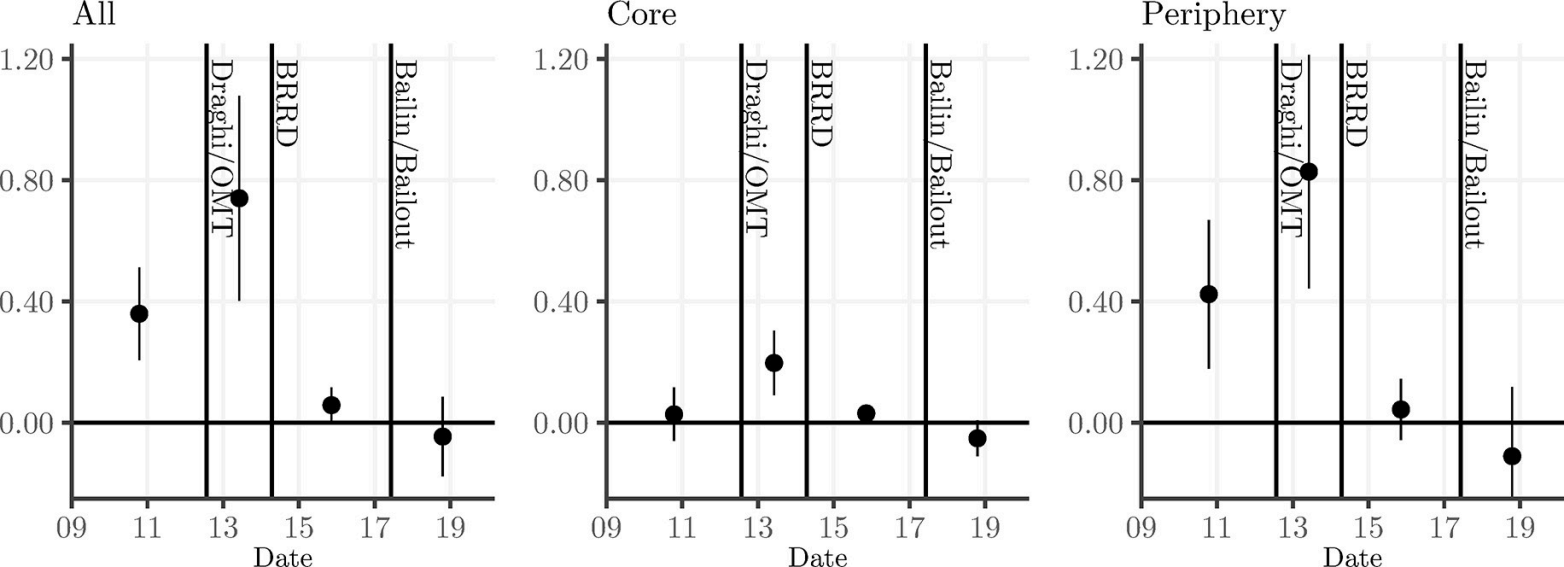
This figure plots the evolution over time of the effect of bank credit risk shocks on sovereign credit risk for the full sample, core and periphery. The vertical axis denotes the size of the beta coefficients of the interactions of the bank shocks with the period dummies. The 5^th^ to 95^th^ percentile confidence interval is plotted as a vertical bar around the estimated beta.

The results in Table 5 reveal a high level of the bank-to-sovereign channel during the great financial crisis and the sovereign debt crisis, especially in the periphery countries (transmission coefficient of 0.42). The transmission did not decrease after ‘Whatever it takes’ and OMT. Hence, the ECB monetary policy actions during the debt crisis were intended to and succeeded in lowering sovereign spreads and this may have affected the sovereign-to-bank transmission of credit risk, but did not affect the bank-to-sovereign risk transmission. On the contrary, the coefficients on the Draghi/OMT interaction terms are positive, indicating a further build-up of bank-to-sovereign contagion in that period. It was only after the BRRD was approved by the parliament in 2014 that the bank to sovereign transmission decreased significantly, in all regions, but in magnitude especially in the periphery countries. The actual bail-in/bailout cases appear to produce only a small downward impact on the bank-sovereign nexus and only significantly so in the core countries. Hence, these cases did not affect the market’s perception of the credibility of the BRRD in the periphery countries. Fig 5 displays the total bank-sovereign transmission in the four regimes we identify, again demonstrating that the enactment of BRRD is the game changer which mitigated the bank-sovereign risk transmission channel. In Fig C.1 in S1 Appendix we plot the evolution of the transmission for each individual country.

**Table 5:** Bank-sovereign risk channel in different regimes (based on Eq (5)). Standard errors in parentheses are clustered at the bank level. *, ** and *** represent significance at the 10%, 5% and 1% percent level, respectively.

	All	Core	Periphery
	Δ*CDS*^*sov*^	Δ*CDS*^*sov*^	Δ*CDS*^*sov*^
Δ*BankRisk*_*i*,*j*,*t*_	0.36∗∗∗	0.03	0.42∗∗
	(0.09)	(0.05)	(0.15)
Δ*BankRisk*×*Draghi*/*OMT*	0.38∗	0.17∗	0.43∗
	(0.20)	(0.09)	(0.23)
Δ*BankRisk*×*BRRD*	-0.68∗∗∗	-0.16∗∗	-0.80∗∗∗
	(0.21)	(0.07)	(0.23)
Δ*BankRisk*×*Bailin*	-0.10	-0.08∗	-0.14
	(0.07)	(0.04)	(0.10)
Bank and Country Controls	Yes	Yes	Yes
Macro Controls	Yes	Yes	Yes
R^2^	0.17	0.04	0.24
Adj. R^2^	0.16	0.03	0.23
No. Obs.	1,543	827	716

Almost simultaneously with the approval of the BRRD, the Single Supervisory Mechanism (SSM) was announced (March 2013) and implemented (November 2014) by the ECB to create a homogeneous supervision of large European banks. [43] report that the announcement of the SSM was associated with reduced contagion between bank and sovereign risk, but that this reduction is determined by the lower influence of sovereign on bank risk after the announcement and not vice versa. To rule out this channel, we include an interaction term of bank risk and the SSM announcement next to the interaction term of bank risk and the BRRD announcement. Table 6 shows the results of this analysis. Our results are robust to this inclusion as the interaction term of bank risk and the announcement of the SSM is insignificant, whereas the interaction term of bank risk and the BRRD announcement is negative and significant, confirming our previous results. While the SSM may have contributed to improving the health of the banking system, we conclude that the main event to lower the bank-to-sovereign risk contagion is the introduction of BRRD.

**Table 6:** Estimation of the bank-to-sovereign risk transmission comparing the SSM period to the BRRD period. Standard errors in parentheses are clustered at the bank level. *** represents significance at the 1% percent level.

	(1)	(2)	(3)	(4)
	Δ*CDS*^*sov*^	Δ*CDS*^*sov*^	Δ*CDS*^*sov*^	Δ*CDS*^*sov*^
Δ*BankRisk*	0.45∗∗∗	0.45∗∗∗	0.42∗∗∗	0.45∗∗∗
	(0.12)	(0.12)	(0.11)	(0.11)
Δ*BankRisk*×*SSM Announcement*	-0.05	-0.05	-0.04	-0.06
	(0.13)	(0.13)	(0.12)	(0.12)
Δ*BankRisk*×*BRRD*_2014_	-0.29∗∗	-0.29∗∗	-0.34∗∗∗	-0.29∗∗
	(0.11)	(0.11)	(0.11)	(0.13)
Bank and Country Controls	No	Yes	Yes	Yes
Macro Controls	No	No	Yes	No
Country and Year FE	No	No	No	Yes
R^2^	0.12	0.12	0.16	0.15
Adj. R^2^	0.11	0.11	0.15	0.14
No. Obs.	1,545	1,537	1,537	1,537

### 5.3 Geographical differences

The intensity of the bank-sovereign risk transmission channel may differ between the core and periphery of the Euro Area or even across countries. One reason may be the varying degree to which banks and sovereigns were affected by the Eurozone debt crisis. Moreover, there is evidence that banks may have used the liquidity injections by the ECB (through LTROs) to increase their exposure to their home sovereign [44] and some banks may have engaged in excessive risk-taking through sovereign exposures [45]. And even after the implementation of the BRRD, it is clear that sovereigns are still able to treat bank recoveries and resolutions with some degree of discretion, e.g. the bail-in of BPoP versus the bailout of Banca MPS. Fig 2 reveals differences not only in the perceived credit risk of banks across the countries in our sample, but also between sovereigns, which might be attributed in part to country-level features which may affect the bank-sovereign nexus. To test for country-specific heterogeneity we estimate Eq (1) for each country in our sample separately, allowing the identification of potential differences in the transmission channel of bank to sovereign credit risk.

The results of the regressions are presented in Table 7 where we match the banks in our sample with their home country (see Table A.1 in S1 Appendix). The coefficients in the first row indicate that the peripheral Euro Area countries, especially Spain and Italy exhibit a pronounced bank-to-sovereign risk channel in the pre-BRRD period and that there were large differences across countries in the transmission of risk before the BRRD. The heterogeneity in the transmission of risk in the period before BRRD is similar to the country results reported in [12]. Countries that were hit hardest during the sovereign debt crisis also experienced the highest impact from the increased risk in the banking sector, further aggravating the stress on these sovereigns, confirming results in [46]. In the period since 2014, with the BRRD approved, the coefficients of risk transmission drop for almost all countries. The introduction of BRRD has thus been beneficial for all, but in magnitude and significance especially for the more vulnerable countries, here Spain and Italy.

**Table 7:** Estimation of the effect of BRRD on the bank-sovereign risk transmission channel (Eq (1)) for each country separately. Standard errors in parentheses are clustered at the bank level. *, ** and *** represents significance at the 10%, 5% and 1% percent level, respectively.

	Netherlands	Italy	Spain	Portugal	France	Germany	Belgium	Austria
	Δ*CDS*^*sov*^	Δ*CDS*^*sov*^	Δ*CDS*^*sov*^	Δ*CDS*^*sov*^	Δ*CDS*^*sov*^	Δ*CDS*^*sov*^	Δ*CDS*^*sov*^	Δ*CDS*^*sov*^
Δ*BankRisk*	0.01	0.76∗∗∗	0.60∗∗∗	0.25	0.05	0.06	0.26	-0.09
	(0.12)	(0.21)	(0.15)	(0.17)	(0.12)	(0.05)	(0.12)	(0.09)
Δ*BankRisk*×*BRRD*	-0.13	-0.79∗∗	-0.56∗∗	-0.18	0.06	-0.06	-0.34	0.13
	(0.12)	(0.27)	(0.18)	(0.15)	(0.14)	(0.05)	(0.20)	(0.07)
Bank and Country Controls	Yes	Yes	Yes	Yes	Yes	Yes	Yes	Yes
Macro Controls	Yes	Yes	Yes	Yes	Yes	Yes	Yes	Yes
R^2^	0.07	0.39	0.4	0.16	0.05	0.05	0.27	0.11
Adj. R^2^	0.01	0.37	0.39	0.08	0.01	0.02	0.18	0.06
No. Obs.	129	312	317	87	196	306	66	130

Table 8 shows whether or not the bank-sovereign nexus still exists after the introduction of the BRRD, estimating Eq (3) for each country separately. The results imply that in all countries there is no longer a transmission of credit risk from banks to their sovereign after the introduction of the BRRD. We show the results graphically in Fig C.1. in the S1 Appendix.

**Table 8:** Estimation of the bank-sovereign transmission before and during the BRRD (Eq (3)) for each country separately. The remaining bank-to-sovereign risk transmission is assessed by the interaction of the bank risk shock and the BRRD time dummy. Standard errors in parentheses are clustered at the bank level. *, ** and *** represents significance at the 10%, 5% and 1% percent level, respectively.

	Netherlands	Italy	Spain	Portugal	France	Germany	Belgium	Austria
	Δ*CDS*^*sov*^	Δ*CDS*^*sov*^	Δ*CDS*^*sov*^	Δ*CDS*^*sov*^	Δ*CDS*^*sov*^	Δ*CDS*^*sov*^	Δ*CDS*^*sov*^	Δ*CDS*^*sov*^
Δ*BankRisk*×*Pre*‒*BRRD*_*t*_	0.01	0.76∗∗∗	0.60∗∗∗	0.23	0.05	0.06	0.26	-0.09
	(0.13)	(0.21)	(0.15)	(0.15)	(0.12)	(0.05)	(0.12)	(0.09)
Δ*BankRisk*×*BRRD*_*t*_	-0.11∗∗	-0.03	0.04	0.06	0.11	0.00	-0.09	0.04
	(0.03)	(0.09)	(0.05)	(0.11)	(0.06)	(0.03)	(0.04)	(0.02)
Bank and Country Controls	Yes	Yes	Yes	Yes	Yes	Yes	Yes	Yes
Macro Controls	Yes	Yes	Yes	Yes	Yes	Yes	Yes	Yes
R^2^	0.07	0.38	0.40	0.13	0.04	0.04	0.27	0.11
Adj. R^2^	0.02	0.37	0.39	0.07	0.01	0.02	0.20	0.07
No. Obs.	129	312	317	87	196	306	66	130

### 5.4 Bank characteristics

Finally, we address the concern that bank-specific factors may affect the channel of risk transmission from bank to the sovereign. We consider two bank characteristics: capital adequacy and systemic size. Better capitalized banks may be more resilient when confronted with negative shocks and exhibit lower risk transmission intensity to their sovereign. Since larger banks would be harder to bailout for governments, the interconnectedness of such banks with their sovereign may be more pronounced. We consider not bank size as such, but rather systemic importance, captured by their G-SIB status, i.e. the additional capital buffer a bank needs to maintain given its classification in one of the G-SIB buckets. To assess the impact of these factors on the impact of the BRRD, we extend Eq (1) to include interactions with the bank capital and bank G-SIB status. In a similar vein, countries with a high debt/GDP ratio may be more vulnerable to bank risk shocks since high debt could impede their capacity to rescue ailing domestic banks. However, including the countries’ debt/GDP ratios with interaction terms yields no significant coefficients.

The hypothesis that better capitalized banks exhibit lower transmission to sovereign credit risk in the BRRD era is tested in Table 9 but receives no support.

**Table 9:** This table presents the results when extending Eq (1) with an interaction of the bank shock with the capital ratio of the bank. Standard errors in parentheses are clustered at the bank level. *** represents significance at the 1% percent level.

	All	Core	Periphery
	Δ*CDS*^*sov*^	Δ*CDS*^*sov*^	Δ*CDS*^*sov*^
Δ*BankRisk*_*i*,*j*,*t*_	0.40∗∗∗	0.05	0.60∗∗∗
	(0.10)	(0.06)	(0.10)
Δ*BankRisk*×*BRRD*	-0.40∗∗∗	-0.04	-0.63∗∗∗
	(0.10)	(0.06)	(0.11)
Δ*BankRisk*×*Capital*	0.02	0.00	-0.12
	(0.05)	(0.02)	(0.08)
Δ*BankRisk*×*BRRD*×*Capital*	0.02	0.00	0.15∗
	(0.05)	(0.03)	(0.08)
Bank and Country Controls	Yes	Yes	Yes
Macro Controls	Yes	Yes	Yes
R^2^	0.16	0.04	0.24
Adj. R^2^	0.16	0.02	0.23
No. Obs.	1,537	823	714

Using the G-SIB buffer of banks as a measure of bank systemic nature, Table 10 shows that prior to the introduction of the BRRD, banks with higher G-SIB buffers in the periphery exhibit a more pronounced transmission of bank shocks to the sovereign (a significant coefficient of 0.83). After the BRRD was voted into law by European Parliament in 2014, the risk transmission for banks in the periphery with G-SIB status decreased more significantly compared to non-G-SIB banks (-0.74). This corroborates the assumption that banks which are more systemic display a more pronounced reduction of the transmission of exogenous bank shocks towards sovereign credit risk, but only in the Euro Area periphery. However, we do not want to stress this finding too much, since when we use the banks’ assets-to-GDP ratio as an alternative indicator of systemic importance, no significant coefficients are found.

**Table 10:** This table presents the results when extending Eq (1) with an interaction of the bank shock with the G-SIB buffer of the bank. Standard errors in parentheses are clustered at the bank level. *, ** and *** represent significance at the 10%, 5% and 1% percent level, respectively.

	All	Core	Periphery
	Δ*CDS*^*sov*^	Δ*CDS*^*so*^	Δ*CDS*^*sov*^
Δ*BankRisk*_*i*,*j*,*t*_	0.38∗∗∗	0.08	0.39∗∗
	(0.10)	(0.05)	(0.14)
Δ*BankRisk*×*BRRD*	-0.33∗∗∗	-0.06	-0.37∗∗∗
	(0.09)	(0.05)	(0.12)
Δ*BankRisk*×*G*‒*SIB*	0.29	0.00	0.83∗∗∗
	(0.27)	(0.06)	(0.14)
Δ*BankRisk*×*BRRD*×*G*‒*SIB*	-0.32	0.01	-0.74∗∗∗
	(0.24)	(0.07)	(0.20)
Bank and Country Controls	Yes	Yes	Yes
Macro Controls	Yes	Yes	Yes
R^2^	0.16	0.03	0.24
Adj. R^2^	0.15	0.02	0.23
No. Obs.	1,295	690	605

## 6. Conclusion

Since 2008 European banks and sovereigns have been confronted with several periods of stress. During the banking crisis and the sovereign debt crisis, some governments undertook bailouts of banks to safeguard the financial sector. In order to avoid the doom loop between banks and sovereigns, the European Banking Union was established, consisting of three pillars: a Single Supervisory Mechanism (with the ECB in the lead), a unified recovery and resolution framework regulated by the Bank Recovery and Resolution Directive, and a European deposit insurance mechanism.

The objective of this paper is to assess the effectiveness of the second pillar of the banking union, i.e. the BRRD regulation. Theoretically, if the bail-in framework introduced by the BRRD is considered credible by market participants, the probability of bank bailouts should decrease and we should observe a significant decrease in the interconnectedness between banks and their sovereign. For our analysis, the bank-to-sovereign transmission is the channel of interest. If the introduction of the BRRD is viewed by the CDS market as credible, unanticipated shocks to the default risk of banks should not, or at least to a much lower extent, be transmitted to the credit risk of the sovereign. This is our motivation to empirically investigate whether or not the implementation of the BRRD has diminished the transmission of bank credit risk to sovereign credit risk, captured with bank and sovereign CDS spreads.

To investigate the effect of changes in perceived bank risk to sovereign credit risk, we identify a series of exogenous bank shocks by considering the change of the banks’ CDS spreads on earnings announcement days, i.e. days on which information on the banks’ risk profile is released. This allows us to isolate the bank-to-sovereign risk transmission channel, which is the relevant channel if the BRRD bail-in framework is considered a credible mechanism to deal with distressed banks. We perform the analysis for 43 banks in 8 Euro Area countries.

Our main findings can be summarized as follows. The results demonstrate that overall, during the period 2009–2020, the transmission channel of bank-to-sovereign credit risk has decreased. More specifically, during the sovereign debt crisis, there was a clear channel from bank to sovereign credit risk, attributed to multiple bank bailouts. After the sovereign debt crisis, the parliamentary approval of the BRRD succeeded in diminishing this transmission channel even to the point of insignificance. This evidence is consistent with the hypothesis that markets consider the BRRD bail-in framework as credible. With respect to country-level heterogeneity, we find that the bank-to-sovereign credit risk transmission channel varies across countries. Yet, in the period after the approval of the BRRD, we find that bank-to-sovereign risk transmission diminishes in most countries, but the mitigating effect is most pronounced for the peripheral countries, especially Italy and Spain. In terms of bank heterogeneity, the only dimension which yields significant results is the systemic nature of the banks, i.e. the higher the G-SIB buffer of the banks, the more the bank-sovereign risk transmission channel diminishes in Euro Area periphery countries, indicating that the additional capital buffer for too-big-to-fail banks imposed by the Basel 3 rules contributes to diminishing the bank-to-sovereign risk transmission. Combined, our findings demonstrate that the BRRD regulation succeeds in diminishing the transmission of bank credit risk to sovereign risk and, hence, alleviates the bank-sovereign doom loop.

## Supporting information

S1 Appendix (DOCX)
