# Supplementary material for: Does BRRD mitigate the bank-to-sovereign risk channel?
Source: PLoS One. 2024 Apr 16;19(4):e0292040. doi: 10.1371/journal.pone.0292040 (PMC11020382; doi:10.1371/journal.pone.0292040)

**Appendix**

**A List of banks in sample**

|  | Bank | Country | Total Assets | as % of GDP | Earnings Announcements |
| --- | --- | --- | --- | --- | --- |
| 1 | Erste Group Bank | Austria | 245,692.85 | 64.89 | 46 |
| 2 | Raiffeisen Bank International | Austria | 152,199.50 | 40.20 | 35 |
| 3 | Raiffeisen Zentralbank Österreich | Austria | 134,846.58 | 36.49 | 19 |
| 4 | UniCredit Bank Austria | Austria | 101,662.60 | 26.85 | 30 |
| 5 | BNP Paribas Fortis | Belgium | 313,195.00 | 68.55 | 22 |
| 6 | KBC Bank | Belgium | 290,591.00 | 63.60 | 44 |
| 7 | Banque Fédérative du Crédit Mutuel | France | 569,947.00 | 24.77 | 10 |
| 8 | BNP Paribas | France | 2,164,713.00 | 94.09 | 47 |
| 9 | Crédit Agricole | France | 1,767,643.00 | 76.83 | 47 |
| 10 | Natixis | France | 496,754.00 | 21.59 | 45 |
| 11 | Société Générale | France | 1,356,495.00 | 58.96 | 47 |
| 12 | Bayerische Landesbank AöR | Germany | 225,965.00 | 6.72 | 45 |
| 13 | Commerzbank | Germany | 463,450.00 | 13.79 | 46 |
| 14 | Deutsche Bank | Germany | 1,297,674.00 | 38.62 | 46 |
| 15 | Hamburg Commercial Bank | Germany | 47,712.00 | 1.42 | 39 |
| 16 | IKB Deutsche Industriebank | Germany | 16,132.00 | 0.48 | 33 |
| 17 | Landesbank Baden-Württemberg | Germany | 256,667.00 | 7.64 | 39 |
| 18 | Portigon | Germany | 4,273.29 | 0.13 | 23 |
| 19 | UniCredit Bank | Germany | 303,598.00 | 9.04 | 35 |
| 20 | Banca Monte dei Paschi di Siena | Italy | 132,196.01 | 8.00 | 45 |
| 21 | Banca Nazionale del Lavoro | Italy | 81,203.00 | 4.52 | 11 |
| 22 | Banca Popolare di Milano Società per Azioni | Italy | 51,131.04 | 2.94 | 32 |
| 23 | Banco BPM | Italy | 167,038.20 | 10.11 | 13 |
| 24 | Banco Popolare Società Cooperativa | Italy | 117,411.00 | 6.75 | 30 |
| 25 | Intesa Sanpaolo | Italy | 816,102.00 | 49.39 | 47 |
| 26 | Mediobanca - Banca di Credito Finanziario | Italy | 82,459.06 | 4.99 | 46 |
| 27 | UniCredit | Italy | 855,647.00 | 51.79 | 46 |
| 28 | Unione di Banche Italiane | Italy | 126,615.99 | 7.66 | 42 |
| 29 | ABN AMRO Group | Netherlands | 375,054.00 | 46.88 | 40 |
| 30 | Coöperatieve Rabobank | Netherlands | 590,598.00 | 73.83 | 23 |
| 31 | ING Bank | Netherlands | 891,910.00 | 111.49 | 45 |
| 32 | NIBC Bank | Netherlands | 22,375.00 | 2.80 | 23 |
| 33 | Banco Comercial Português | Portugal | 81,643.41 | 40.80 | 47 |
| 34 | Banco Espírito Santo | Portugal | 80,608.02 | 46.58 | 20 |
| 35 | Novo Banco | Portugal | 45,295.90 | 22.64 | 20 |
| 36 | Banco Bilbao Vizcaya Argentaria | Spain | 695,471.00 | 61.99 | 48 |
| 37 | Banco de Sabadell | Spain | 223,753.64 | 19.94 | 47 |
| 38 | Banco Popular Español | Spain | 147,685.80 | 12.71 | 30 |
| 39 | Banco Santander | Spain | 1,522,695.73 | 135.72 | 46 |
| 40 | Bankia | Spain | 208,468.27 | 18.58 | 34 |
| 41 | Bankinter | Spain | 83,732.35 | 7.46 | 48 |
| 42 | Caixa Geral de Depósitos | Spain | 85,776.06 | 7.65 | 35 |
| 43 | CaixaBank | Spain | 391,413.97 | 34.89 | 29 |

**Table A.1:** Overview of the bank sample. This table shows the banks included in our sample together with the country where they are headquartered. In the third column we show the most recent observation of total assets in millions in our sample period, whilst in the fourth column we display the size of the bank relative to the GDP of their home country. The final column shows the number of earnings announcements per bank.

**B Robustness: 2-day event window**

|  | (1) | (2) | (3) |
| --- | --- | --- | --- |
|  | ${\Delta CDS}^{sov}$ | ${\Delta CDS}^{sov}$ | ${\Delta CDS}^{sov}$ |
| ${\Delta BankRisk}_{i,j,t}$ | 0.29∗∗∗ | 0.29∗∗∗ | 0.27∗∗∗ |
|  | (0.09) | (0.09) | (0.09) |
| ${\Delta BankRisk}\times{BRRD}$ | -0.16∗ | -0.17∗ | -0.20∗∗ |
|  | (0.09) | (0.09) | (0.08) |
| Bank and Country Controls | No | Yes | Yes |
| Macro Controls | No | No | Yes |
| R^2^ | 0.09 | 0.09 | 0.12 |
| Adj. R^2^ | 0.09 | 0.09 | 0.11 |
| No. Obs. | 1,534 | 1,532 | 1,532 |

**Table B.1:** This table presents the results from estimation of Equation (1), where the shock is defined in a 2 day event window. The transmission of bank-to-sovereign credit risk is significantly reduced after the introduction of BRRD regulation. Standard errors in parentheses are clustered at the bank level. *, ** and *** represent significance at the 10%, 5% and 1% percent level, respectively.

**C Country and time heterogeneity**

**Figure C.1:** This figure plots the evolution over time of the effect of bank credit risk shocks on sovereign credit risk for each country individually. The vertical axis denotes the size of the beta coefficients of the interactions of the bank shocks with the year dummies. The 5th to 95th percentile confidence interval is plotted as a vertical bar around the estimated beta.


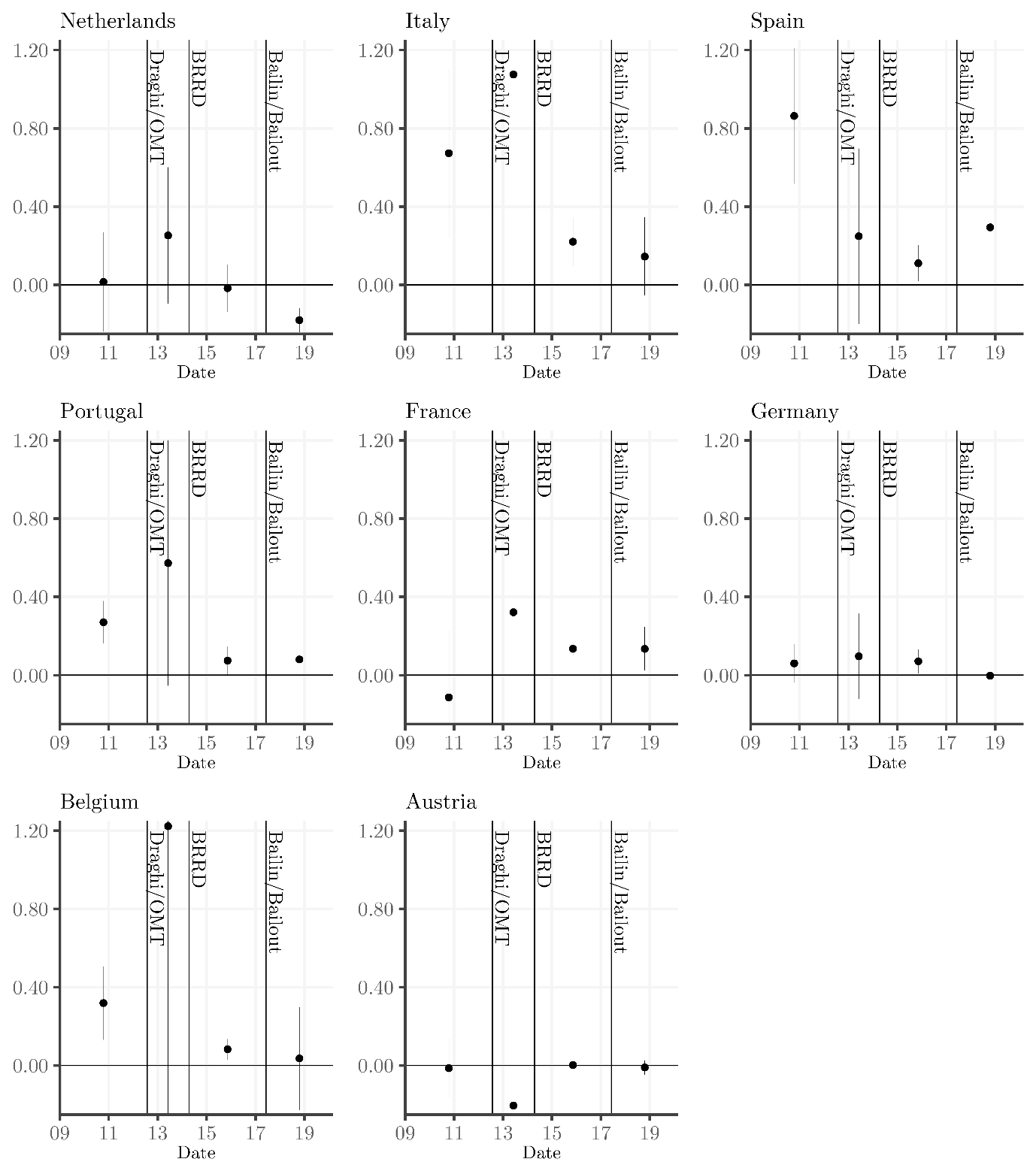

Supplement: S1 Appendix — (DOCX) [file pone.0292040.s001.docx]
